# The effects of rear-wheel camber on the kinematics of upper extremity during wheelchair propulsion

**DOI:** 10.1186/1475-925X-11-87

**Published:** 2012-11-22

**Authors:** Chung-Ying Tsai, Chien-Ju Lin, Yueh-Chu Huang, Po-Chou Lin, Fong-Chin Su

**Affiliations:** 1 Department of Biomedical Engineering, National Cheng Kung University, 1 University Road, Tainan City 701, Taiwan; 2 Medical Device Innovation Center, National Cheng Kung University, 1 University Road, Tainan City 701, Taiwan

**Keywords:** Wheelchair, Camber, Kinematics

## Abstract

**Background:**

The rear-wheel camber, defined as the inclination of the rear wheels, is usually used in wheelchair sports, but it is becoming increasingly employed in daily propulsion. Although the rear-wheel camber can increase stability, it alters physiological performance during propulsion. The purpose of the study is to investigate the effects of rear-wheel cambers on temporal-spatial parameters, joint angles, and propulsion patterns.

**Methods:**

Twelve inexperienced subjects (22.3±1.6 yr) participated in the study. None had musculoskeletal disorders in their upper extremities. An eight-camera motion capture system was used to collect the three-dimensional trajectory data of markers attached to the wheelchair-user system during propulsion. All participants propelled the same wheelchair, which had an instrumented wheel with cambers of 0°, 9°, and 15°, respectively, at an average velocity of 1 m/s.

**Results:**

The results show that the rear-wheel camber significantly affects the average acceleration, maximum end angle, trunk movement, elbow joint movement, wrist joint movement, and propulsion pattern. The effects are especially significant between 0° and 15°. For a 15° camber, the average acceleration and joint peak angles significantly increased (p < 0.01). A single loop pattern (SLOP) was adopted by most of the subjects.

**Conclusions:**

The rear-wheel camber affects propulsion patterns and joint range of motion. When choosing a wheelchair with camber adjustment, the increase of joint movements and the base of support should be taken into consideration.

## Background

Wheelchairs allow people with disabilities to achieve independent mobility. 51.2 million people in the U.S. have a physical disability [1], and there are about 265,000 people with spinal cord injury (SCI) in U.S. in 2010 [2]. The long-term use of wheelchairs often leads to injuries of the upper extremities. Gellman reported that most wheelchair users (67.8%) complained about pain in at least one area of their upper extremities [3]. For wheelchair users, shoulders and wrists are the major joints suffering from injuries [3-6]. Overuse injuries are commonly seen in wheelchair athletes and they recur more often than do other injuries [7]. Furthermore, in the fatal wheelchair-related accidents, about three quarters of wheelchair users experienced tipping or falling from their wheelchairs [8]. Therefore, improving the stability of wheelchairs and increasing the efficiency of wheelchair propulsion will help reduce injuries.

Studies have been conducted to understand the mechanics of wheelchair propulsion and the findings show that the performance of wheelchair users is affected by factors such as velocity, the surface of the ground, and users’ physical capability. Wheelchair propulsion involves a man–machine system and it has been reported that changes in any part of the wheelchair, such as handrim size, seat height, and wheel axel position, alter propulsion patterns, applied forces, physiological parameters, and mechanical efficiency [9-14].

The rear-wheel camber, which is the inclination of the rear wheels, is usually used in wheelchair sports, but it is becoming increasingly employed in daily propulsion. The advantages of a camber include a decrease in the down turning moment when the wheelchair is on a lateral slope [15], lower stress on the bearings when the wheelchair turns at high speed [16], hand protection and comfort, as well as crash prevention during games [16,17], increased turning velocity, and improved lateral stability [17,18]. The disadvantages include a larger wheelbase which could impede passage through narrow doorways, and increased strain on the wheel ball-bearings [16]. The selection of a proper rear-wheel camber is thus important for wheelchair users.

The effects of camber have been evaluated by several researchers. Veeger et al. examined the effects of various camber angles (0°, 3°, 6°, and 9°) on wheelchair propulsion, and found no kinematical or physiological benefits, such as lowering oxygen cost and mechanical efficiency, as well as changing push angle and time, to using a camber [16]. Their study indicated that the rolling resistance decreased with increasing camber. Perdios’ study also shows that cambers (0°, 3°, and 6°) will not influence the cardiopulmonary variables during wheelchair propulsion [17]. However, the opposite was found in the study of Faupin et al., who concluded that rolling resistance increased when the camber angle was increased from 9° to 15° [19]. They found that when the camber angle was increased, the mean velocity decreased, and both of the power output and the duration of the propulsion phase increased [19]. In the study of Huang et al., they found that when the camber changed from 0° to 15° there was a larger discrepancy between mechanical work and power flow. It shows that the larger camber would cause more energy loss [20]. Similar results were observed in another study with increasing camber from 15° to 24° [14]. Another study indicated that the 24° camber might have negative effects on linear maximal effort mobility performance when compared with 15°, 18°, and 20° camber due to increased resistance, while 18° camber might be a recommended setting for young or inexperienced athletes because of its superior performance for each aspect of mobility performance [21].

In order to increase stability, wheelchair athletes used wheelchairs with cambers during competition, but increasing camber angle may affect physiological parameters during propulsion, especially energy consumption. However, few studies have been conducted on the effects of camber on the kinematics of the upper extremities. The present study thus examines the effects of camber on kinematics during wheelchair propulsion on level ground. The results of the study help demonstrate the effects of wheelchair design on propulsion mechanics.

## Methods

Twelve inexperienced wheelchair users without cardiovascular disease and musculoskeletal disorders in their upper extremities participated in this study, which was approved by our hospital Institutional Review Board. Before testing, all subjects were informed about the study and signed a consent form. The body weight, height, and arm span of each subject was measured prior to testing. The mean values (± standard deviation) of age, body weight, height, and arm span are 22.3±1.6 yr, 72.9±5 kg, 175.4±3.7 cm, and 178.4±5.5 cm, respectively.

**Figure 1:**
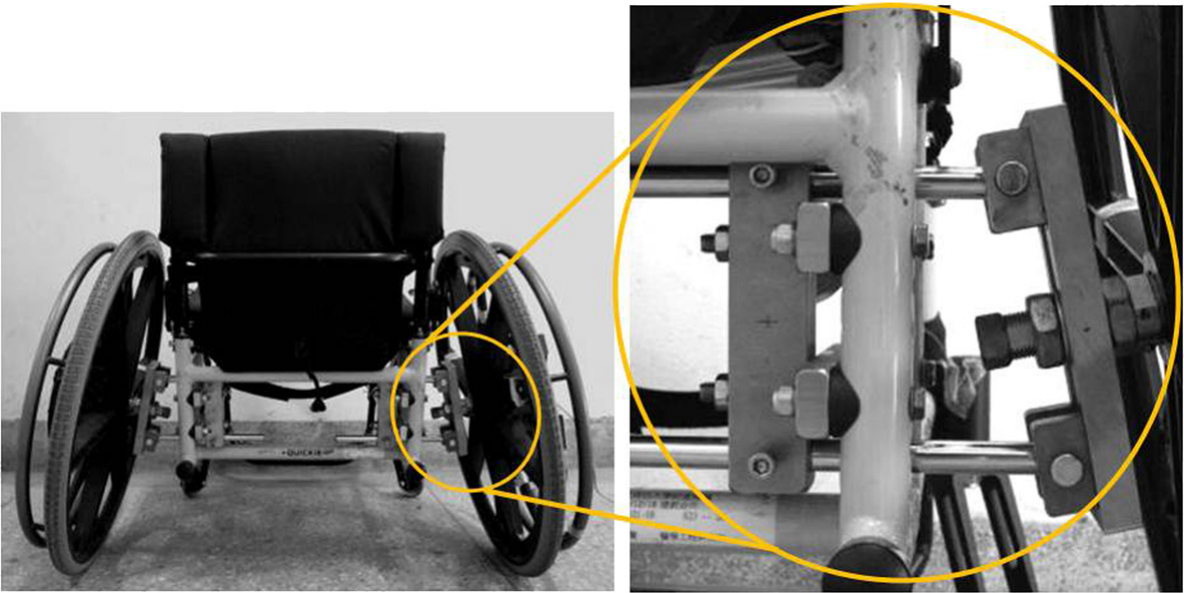
**A self-designed mechanism for rear-wheel camber adjustment. **The mechanism was mounted on the wheelchair to adjust the rear-wheel camber.

A Quickie GP ultralight wheelchair was used by all participants. A custom-designed mechanism mounted on the wheelchair was adopted to adjust the rear-wheel camber and the distance between the tops of the two handrims (Figure 1). A video-tracking system (Motion Analysis Corporation) with eight digital cameras was used to collect the three-dimensional trajectories of the markers that were attached to the trunk, upper arm, fore-arm, hand, and wheel at a sampling rate of 60 Hz. One instrumental wheel, consisting of a six-component load cell (Model UFS-4515A, JR3, Inc., Woodlanc), was mounted on the wheelchair to collect the hand-rim contact force and moment [22].

Three cambers (0°, 9°, and 15°) were tested in this study. The camber of 15° is often used in the wheelchair basketball game [19]. In order to minimize the effects of the anthropometric differences between participants, the distance between the tops of the handrims was adjusted according to their arm spans (the distance was equal to 40% of arm span, as used for conventional width of a wheelchair or doorway [23]) and maintained the same distance in the three cambers. To prevent toe in or toe out, all the rear wheels were carefully adjusted to maintain the proper wheel alignment [14]. A granular canvas was paved to simulate outdoor wheelchair propulsion. Fourteen reflective markers were attached to the following places on the trunk and right upper extremity: the spinous process of the 7th cervical vertebrae, the sternal notch, the right acromion process, the xiphoid process, the lateral epicondyle, the medial epicondyle, the radial styloid process, the ulnar styloid process, the head of the 2nd metacarpal bone, the head of 5th metacarpal bone, and the middle shaft of the 3rd metacarpal bone. A triangular frame with three markers was attached to the lateral side of the upper arm. An additional five markers attached to the wheel were used to determine the coordination of the wheel frame. Because wheelchair users’ propulsion movement is similar between right and left sides in an indoor and laboratory condition [24], plus reduce the influence of markers, the study collected both the kinematic and kinetic data on the right side.

Before data collection, subjects were allowed to practice wheelchair propulsion to become acclimated to the wheelchair. During the experiments subjects were asked to propel the wheelchair at an average velocity of 1 m/s for three cambers angles (0°, 9°, and 15°). For each condition consisting of five successful trials, defined as stably propelling the wheelchair through a 4-meters pathway in 4 seconds, were recorded. The test conditions were changed in random order to minimize the effects of fatigue.

One complete propulsion phase is determined by initial and end of hand-rim contact force detected by the instrumental wheel. Prior to the kinematic modeling of the upper extremity, the three-dimensional trajectories data were filtered with a generalized cross validation spline smoothing routine with a cutoff frequency of 6 Hz. The kinematics model of the upper extremity established by Guo et al. [25] was used, in which the upper extremity was assumed to be a four-segment-linkage system (trunk, upper arm, forearm, and hand). Each segment was treated as a rigid body. Joint movement is described using the orientation of a distal segment coordinate system relative to a proximal segment coordinate system. The local coordinate system for each segment was aligned in the same direction and defined with the positive x-axis toward subjects’ backward, positive y-axis toward subjects’ right, and the positive z-axis orthogonal to x- and y-axis, pointing superior. Euler angles are used to describe the orientation of joint movements [26]. The rotation sequence chosen was y-x’-z”. The glenohumeral rotation center was determined by a constant offset method which estimates the rotation center using constant distance ratio relative to acromion marker and the mid-point between lateral and medial epicondyle markers [27].

**Figure 2:**
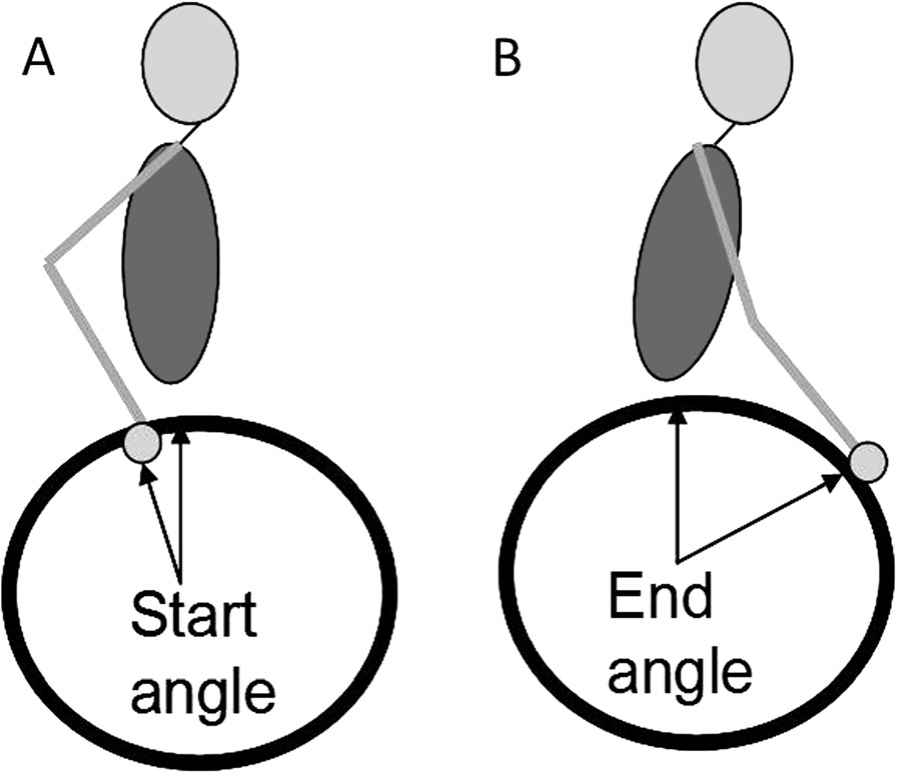
**Definitions of start angle and end angle. **(**A**) The start angle is the angle between the line which connects the initial hand-rim contact point of the propulsion phase to the wheel center and the vertical line. (**B**) The end angle is the angle between the line which connects the final hand-rim contact point of the propulsion phase to the wheel center and the vertical line.

The kinematic parameters including peak joint angle and joint range of motion (ROM), and propulsion pattern were analyzed. The definition of propulsion pattern was based on the study of Boninger et al. [28]. Semicircular pattern is recognized by hand falling down and back to the starting point during recovery phase. For the single loop (SLOP) pattern, during the recovery phase, the hand rises above the hand-rim. For the double loop (DLOP) pattern, the hand performs a double loop movement which rises above the hand-rim and crosses over during the recovery phase. For the arcing pattern, the hand moves along the same path in both of the propulsion phase and the recovery phase. The temporal-spatial parameters, including average acceleration, maximum start angle, maximum end angle, and maximum stroke angle, were examined. Average acceleration was computed from the acceleration of the wheelchair, which was measured from the minimum velocity to the maximum velocity in one propulsion cycle. The start angle (Figure 2A) is the angle between the line which connects the initial hand-rim contact point of the propulsion phase to the wheel center and the vertical line. The end angle (Figure 2B) is the angle between the line which connects the final hand-rim contact point of the propulsion phase to the wheel center and the vertical line. The stroke angle is the sum of the start angle and the end angle. The maximum start, end, and stroke angles are the maximum values obtained from all cycles in one trial.

The repeated measures analysis of parameters was performed using the SPSS 13.0 software package. The differences of variables were compared for the three cambers. When the results of repeated measures analysis were significant, the least significant difference (LSD) pair-wise multiple comparison test was used as post hoc to find the specific groups which had the significant difference. The p-value was set at 0.05 as the level of significance.

## Results

### Temporal-spatial parameters

The results of the temporal-spatial parameters of wheelchair propulsion are listed in Table 1. The camber significantly affected the average acceleration and maximum end angle. When the camber was increased from 0° to 15°, the average acceleration and the maximum end angle significantly increased.

**Table 1:** Results of temporal-spatial parameters for the three camber angles

	**0°**	**9°**	**15°**	**Sig. (p)**
**Stroke frequency (1/s)**	1.02±0.19	1.02±0.20	1.11±0.18	0.28
**Average acceleration (m/s**^**2**^**)**	0.41±0.06	0.43±0.07	0.55±0.12	†0.000
				0°<15°; 9°<15°
**Max. end angle (°)**	58.75±7.94	61.83±8.67	63.08±7.17	*0.013
				0°<15°
**Max. start angle (°)**	16.39±12.25	15.38±10.34	14.09±8.49	0.46
**Max. stroke angle (°)**	75.14±6.54	77.35±7.12	77.37±6.09	0.421

Note. Values are mean ± SD.

* p < 0.05.

† p < 0.01.

### Maximum range of motion and peak angle

Table 2 shows the maximum ROMs and peak angles of the trunk for the three cambers. The maximum ROM of the trunk flexion/extension increased with increasing camber. No significant difference was found in the maximum ROMs between the 0° and 9° cambers. The peak angle of the trunk flexion for the 15° camber was significantly larger than those of the 0° and 9° cambers. There was no significant difference in the peak angles of the trunk extension between the three cambers.

**Table 2:** Results of the ROM of the trunk and peak angles for the three camber angles

**Trunk**	**0°**	**9°**	**15°**	**Sig. (p)**
**Flexion/extension ROM (°)**	6.83±1.99	7.72±2.69	10.36±3.13	†0.002
				0°<15°; 9°<15°
**Peak flexion angle(°)**	9.14±11.28	10.53±10.93	14.97±10.53	*0.029
				0°<15°; 9°<15°
**Peak extension angle(°)**	2.06±10.25	2.55±8.70	4.92±10.27	0.302

Note. Values are mean ± SD.

* p < 0.05.

† p < 0.01.

Abbreviations: ROM, range of motion.

**Figure 3:**
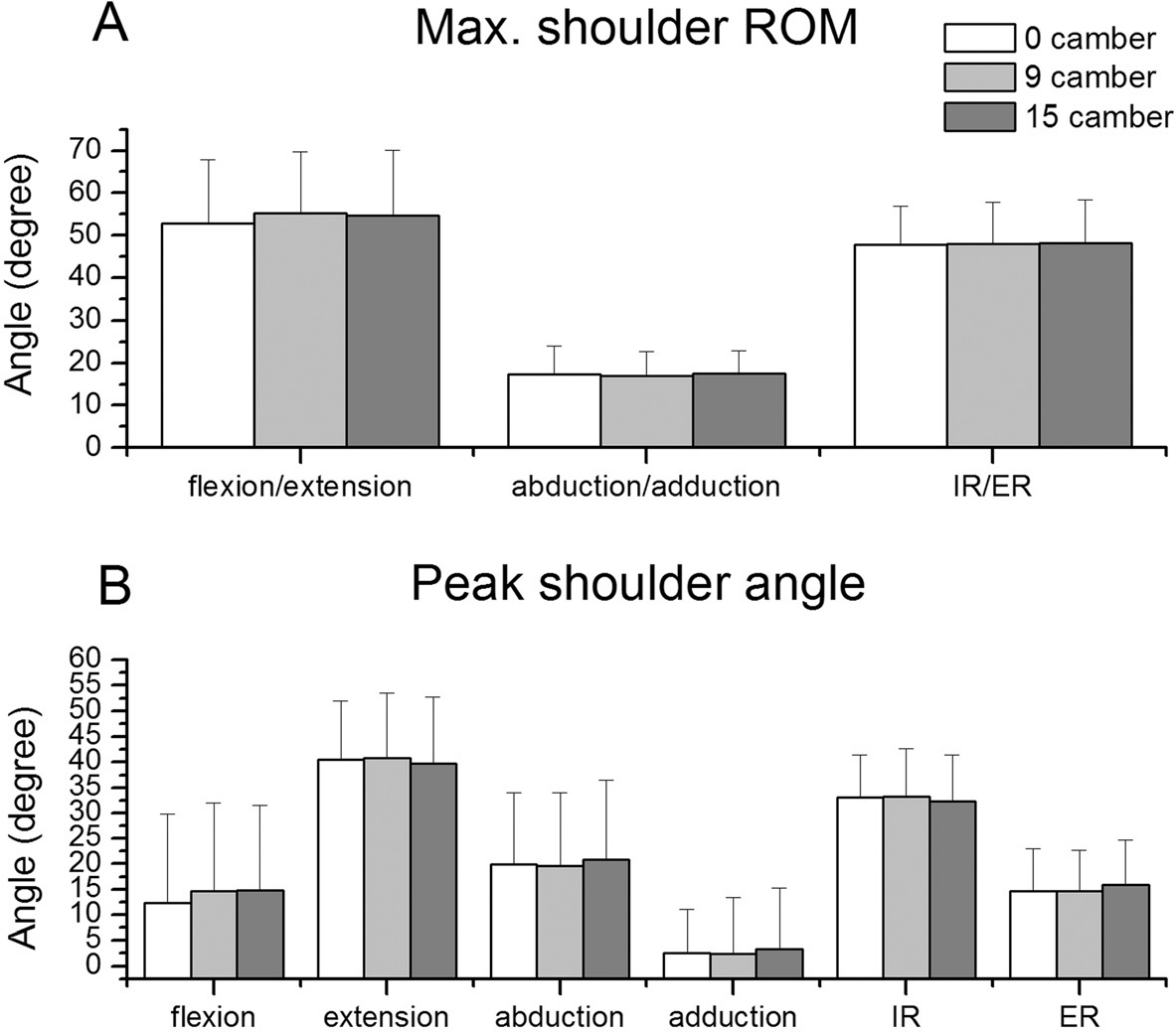
**Results of shoulder kinematics. **(**A**) Maximum shoulder range of motion and (**B**) peak shoulder angle for three cambers. Abbreviations: ROM, range of motion; IR, internal rotation; ER, external rotation.

Results of the maximum ROMs and peak angles of the shoulder joint are shown in Figure 3. The maximum ROMs of the shoulder flexion/extension, abduction/adduction, and internal/external rotation ranged over 52.81°-55.31°, 17.02°-17.48°, and 47.84°-48.27°, respectively. There was no significant difference in either maximum ROMs or peak angles of the shoulder joint between the three cambers.

**Figure 4:**
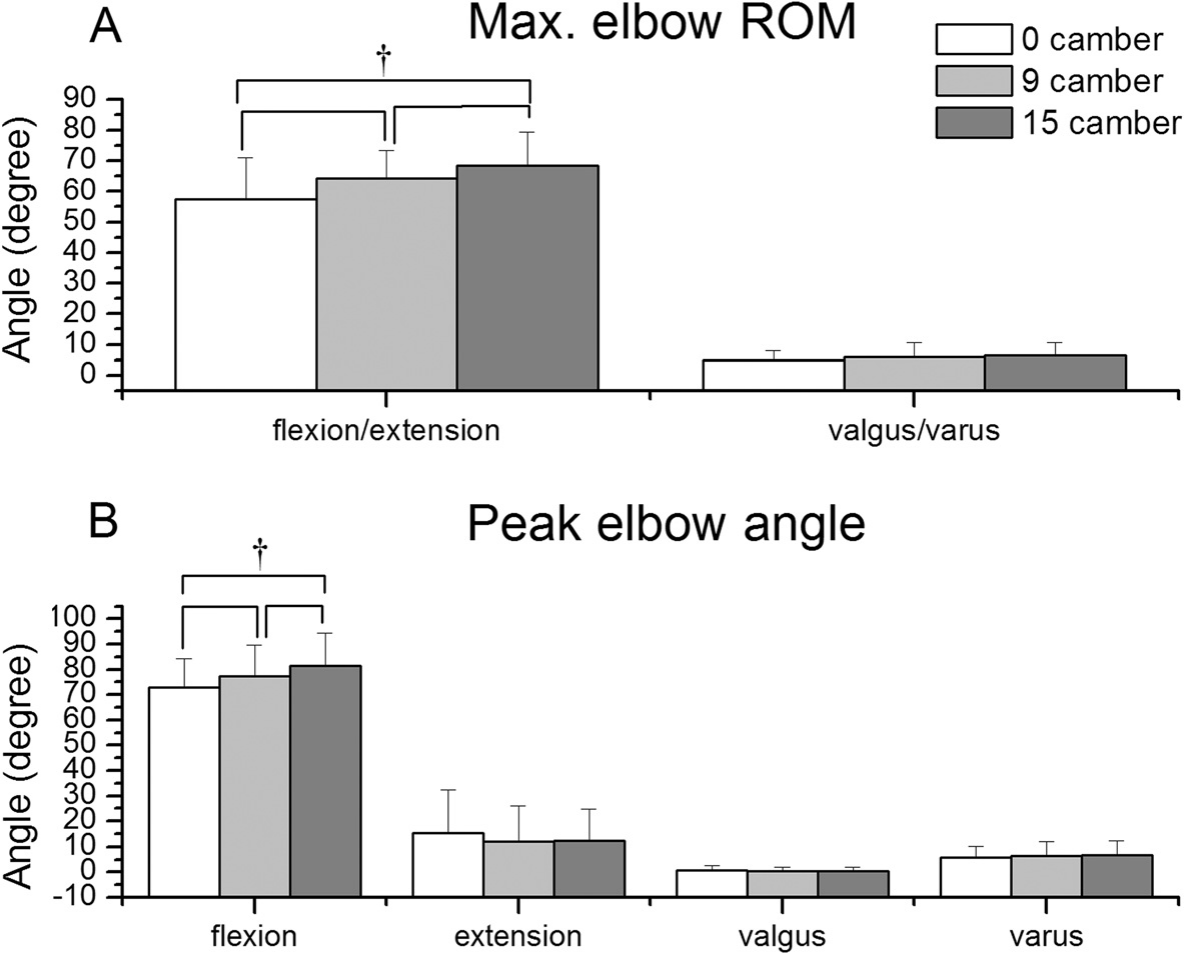
**Results of elbow kinematics. **(**A**) Maximum elbow range of motion and (**B**) peak elbow angle for the three camber angles. Note. † p<0.01.

The maximum ROM and peak angles of the elbow joint are illustrated in Figure 4. When the wheelchair with three different cambers, respectively, was propelled, the maximum ROMs of the elbow flexion/extension and valgus/varus ranged over 57.37°-68.35° and 4.95°-6.44°, respectively. The maximum ROM in the elbow flexion/extension increased with increasing camber. A significant difference existed not only between 0° and 15° cambers, but also between 0° and 9° cambers. No significant difference in the maximum elbow valgus/varus ROM was observed between the three cambers.

**Figure 5:**
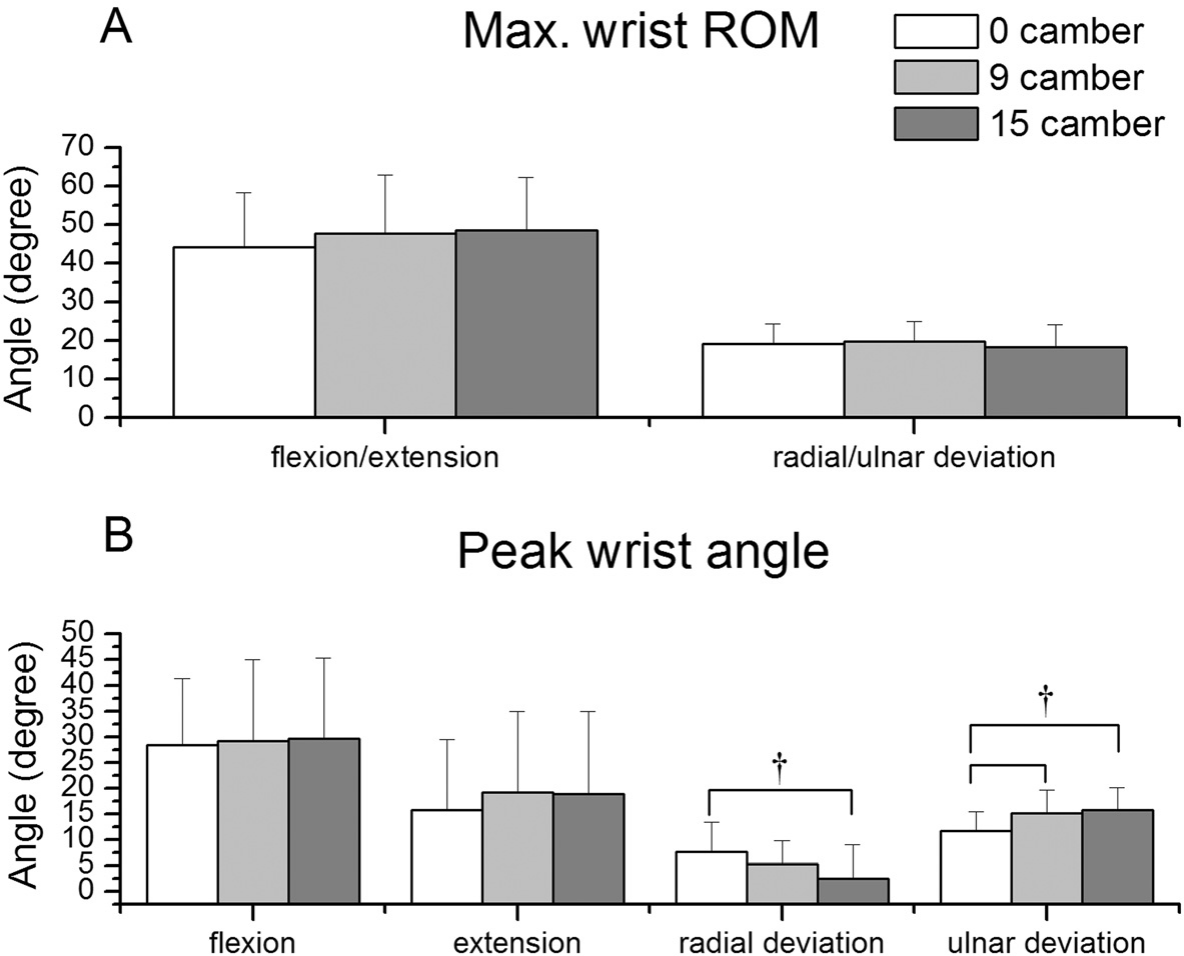
**Results of wrist kinematics. **(**A**) Maximum wrist range of motion and (**B**) peak wrist angle for the three camber angles. Note. † p<0.01.

Figure 5 shows the results of maximum ROM and peak angles of the wrist joint. The maximum ROMs in the wrist flexion/extension and radial/ulnar deviation for the three angles ranged over 44.11°-48.45° and 18.44°-19.79°, respectively. The maximum ROM in neither the flexion/extension nor the radial/ulnar deviation had significant difference between the three angles. Increasing camber resulted in peak angles significantly decreasing in the wrist radial deviation and increasing in the wrist ulnar deviation.

### Propulsion pattern

**Figure 6:**
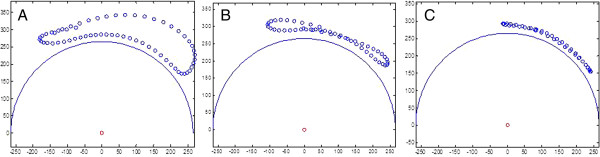
**Propulsion patterns. **(**A**) single loop (SLOP), (**B**) double loop (DLOP), and (**C**) arcing.

Subjects demonstrated three propulsion patterns: single loop (SLOP) (Figure 6A), double loop (DLOP) (Figure 6B), and arcing (Figure 6C). The propulsion patterns used for the three cambers are shown in Table 3. According to the results, when the camber angle was increased from 0° to 15°, subjects preferred to use the SLOP pattern.

**Table 3:** Number of propulsion patterns used for the three camber angles

**Pattern**	**0°**	**9°**	**15°**
**Result**	SLOP:5	SLOP: 8	SLOP: 11
	DLOP:6	DLOP: 4	Arcing:1
	Arcing:1	Arcing:1	

Abbreviations: SLOP, single loop; DLOP, double loop.

### Joint movement pattern

**Figure 7:**
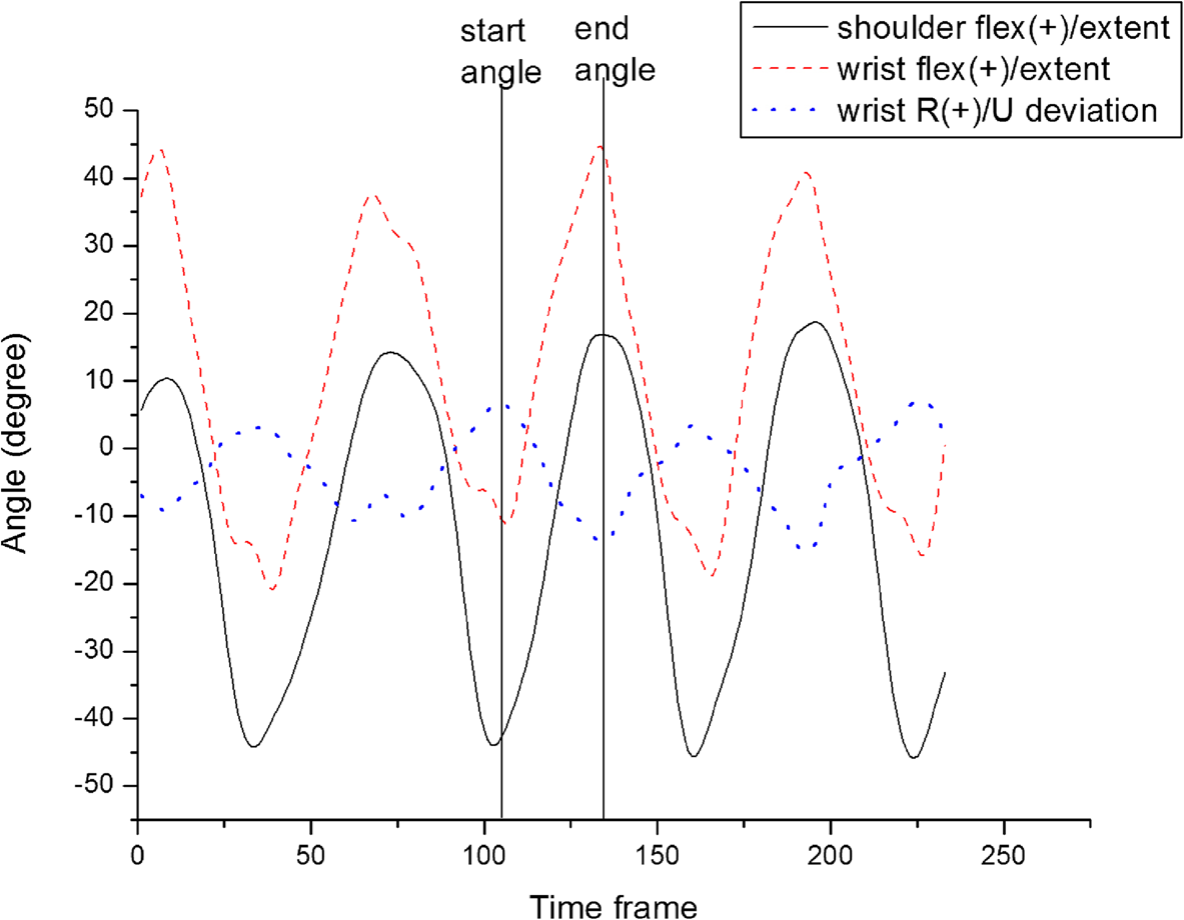
**Movement patterns of shoulder and wrist joints. **During propulsion phase, the shoulder and wrist joints move from extension to flexion and the wrist joint moves from radial deviation to ulnar deviation. (A representative figure from one subject while propelling the wheelchair with 9 degree of camber).

The wrist and shoulder joint movement patterns during wheelchair propulsion are shown in Figure 7. At the start angle the shoulder starts to flex, while the wrist performs extension and radial deviation. At the end angle, at the end of the propulsion phase, the shoulder flexion is at its maximum, while the wrist achieves the maximum flexion and ulnar deviation.

## Discussion

The temporal-spatial parameters, propulsion patterns, and kinematics of the upper extremity during the propulsion of a wheelchair with various rear-wheel cambers were examined in this study. When users tried to propel the wheelchair with the three cambers respectively at the same average velocity, the average acceleration was significantly greater for the 15° camber than those for the 0° and 9° cambers. Faupin et al. discussed the effects of camber during wheelchair sprinting. Their results showed that rolling resistance of a wheelchair increased with increasing camber in the range of 9°-15° [19]. However, Veeger et al. indicated that rolling resistance decreased when the camber was increased from 0° to 9° [16]. Combing the results of these studies, the change of rolling resistance in the camber angle range of 0°-9° is not significant, but increases significantly at 15°. The larger rolling resistance for the 15° camber decreases the velocity of the wheelchair. In order to maintain a stable velocity, the average acceleration of the wheelchair with a 15° camber must increase.

The maximum end angle for the 15° camber was significantly greater than that for the 0° camber. Besides, the angle of trunk forward flexion significantly increases from 0° camber to 15° camber. With increasing camber, subjects tried to change their stroke pattern and trunk movement. This might be due to the average acceleration increasing with greater camber, which made users flex trunk more to facilitate the velocity of their upper extremity and change their propulsion patterns to maintain a stable wheelchair velocity. Similar results were obtained by Vanlandewijck, who found that when the speed of a wheelchair increased, wheelchair users would change their propulsion pattern from pull-push movement to stroke and also change their trunk movement [29].

Changes of propulsion pattern were observed in this study. Most of the subjects adopted SLOP and DLOP patterns with 0° camber. However, with 15° camber, almost all of them changed to SLOP pattern. These results are consistent with those obtained in Boninger’s and de Groot’s study [28,30]. In his study, the two most common propulsion patterns were also SLOP and DLOP. They showed that SLOP might be the most natural pattern. For the SLOP pattern, subjects just need to raise their hand up from the hand-rim. Therefore, most people without wheelchair training would use this kind of pattern. Changing propulsion pattern to SLOP after increasing speed was observed by de Groot’s study [30]. According to de Groot’s and current studies, the increasing speed and camber resulted in a higher workload, so almost all subjects used the SLOP pattern which is the most natural and controllable propulsion to compromise increasing load. However, previous studies have indicated that the semi-circular pattern is best for wheelchair users [28,31]. The accelerations of the shoulder and elbow are lower and propulsion phase is longer for this pattern. Previous studies have also shown that the frequency of propulsion is correlated with the risk of median nerve injury [32]. Therefore, propelling with the semi-circular pattern may decrease the probability of injury. Wheelchair users should adopt the semi-circular pattern when propelling with a large camber to avoid injury.

With regards to the kinematics of the trunk and upper extremities, the movement patterns and ROMs of each joint are comparable with those measured in other studies on the kinematics of wheelchair propulsion [28,29,33].

As for the shoulder ROM and shoulder peak angles, there was no significant difference between the three cambers. The distance between the tops of the two wheels was made the same for the three cambers to minimize its effect. However, the ROM in the elbow flexion/extension, and peak angles in the wrist radial deviation and ulnar deviation changed significantly for the 15° camber. Therefore, the effects of camber on the kinematics of the upper extremity at a leisure velocity begin from the distal segment. The ROMs of the shoulder flexion in the present study were about 50°-55° for the three cambers, which is similar to the findings in Vanlandewijck’s study [29]. They also showed that the shoulder ROM is not affected by the change of speed. Although the muscles around the shoulder joint are thought to be the primary movers during wheelchair propulsion, changing the rear-wheel cambers for the velocity of 1m/s does not influence shoulder movement, instead resulting in major changes in the trunk, elbow, and wrist movements.

The ROMs of elbow flexion/extension and peak angle in the flexion significantly increased with increasing camber. The increase in the trunk flexion for a given set of shoulder joint movements decreased the vertical distance from the shoulder joint to the wheel axis, which increased in the elbow flexion [34]. In addition, the propulsion pattern affects the movements of the elbow joint. Compared with DLOP, the propulsion pattern for SLOP requires more use of the elbow flexion.

Although the ROM of the wrist joint did not increase with increasing camber, the peak ulnar and radial deviation angles have significant difference in 15° of camber. For the 15° camber, the angle in the radial deviation was the lowest, while the angle in the ulnar deviation was the highest. The movement of the wrist is affected by the movement of the trunk and the elbow. The start and end angles also greatly influence the movement of the wrist. From the joint movement pattern at the start angle, the radial deviation of the wrist reached its maximum, while at the end angle, the ulnar deviation of the wrist reached its maximum. Therefore, decreasing of the start angle for the 15° camber decreases the radial deviation of the wrist, and an increase of the end angle increases the ulnar deviation of the wrist. The results are similar with the study of Boninger et al. in 1997 [35]. When the speed increased, wheelchair users’ radial deviation angle significantly decreased. As the loading of propulsion increases it will make users decrease wrist radial deviation.

Veeger analyzed the wrist motion during wheelchair propulsion without a camber on a constant slope [33]. The peak values of the wrist flexion angle, extension angle, radial deviation angle, and ulnar deviation angle in his study were −1±18°, 42±16°, 21±6°, and 24±8°, respectively. These results are different from those obtained in the present study. The differences in the results between these two studies might come from the different experimental parameters, such as the width of the hand-rim, the velocity of wheelchair propulsion, seat position, seat height, and slope.

All of the peak joint angles were within the normal ROMs. Therefore, although a large camber increased the peak joint angle during wheelchair propulsion, the increase of the joint angle might not result in overstretching injuries of the tissues around the joints. Further investigation should be conducted to understand whether the increased ROMs of each joint result in greater joint moments, which could also lead to injuries.

There were limitations in the study. First of all, the subjects in the study were inexperienced wheelchair users. However, recruiting inexperienced wheelchair users as subjects helps minimize the effects of adaptation technique from experienced individuals. They can also be used to simulate new wheelchair users [36]. Too few subjects were tested and therefore increasing the number of subjects will increase the validity and power of the study. The space limit in the laboratory prevented the subjects from propelling the wheelchair for long distances and achieving a stable speed. We increased the number of trials and decided upon a propulsion cycle which was closest to the target speed in each trial in order to negate the fluctuation in speed due to space limitation. Lastly, the alignment of the wheelchair was not maintained after changing camber angle. For example, seat height and tilt angle of a wheelchair frame would be changed. However, compared to these subjects’ height, the differences of these changed parameters in the three cambers are relatively small. They did not affect these users’ movement pattern.

## Conclusions

The optimal design of a wheelchair must consider many parameters, including seat position, hand-rim size, and camber. The designer must consider the user’s needs as well as his or her physical condition when choosing the most suitable components for a wheelchair. The results of this study show that the camber is an important parameter. The effects of camber on the kinematics of the upper extremity include increased joint movement, such as ROM and peak angle of elbow and trunk flexion, as well as wrist ulnar and radial deviation, and changed propulsion pattern to SLOP. Wheelchairs with a rear-wheel camber of less than 9° should be used to increase the stability of wheelchair while maintaining maneuverability for daily activities. The results of this study provide important information for wheelchair users and designers.

## Competing interests

The authors declare that there are no conflicts of interest in this study.

## Authors’ contributions

CYT was involved in the study design, data acquisition and analysis, as well as concluded every author’s interpretation to complete the manuscript. CJL helped in data collection, analysis, and interpretation. She also participated in protocol design and manuscript modification. YCH helped in interpreting the results and revising the manuscript. PCL helped in data collection and analysis, and protocol design. FCS provided the conception and design of the study, guided the data analysis, as well as involved in the final revision of the manuscript. All the authors read and approved the final manuscript.
